# Editorial: Discussing the implication of DNA methylation in human diseases

**DOI:** 10.3389/fmolb.2026.2015661

**Published:** 2026-10-16

**Authors:** Pietro Salvatore Carollo, Andrea Fuso, Viviana Barra

**Affiliations:** 1 Department of Medicine and Surgery, University of Enna “Kore”, Enna, Italy; 2 Department of Experimental Medicine, Sapienza University of Rome, Rome, Italy; 3 Department of Biological, Chemical and Pharmaceutical Sciences and Technologies (STEBICEF), University of Palermo, Palermo, Italy

**Keywords:** cell free DNA (cfDNA), epigenetic clock, DNA methylation, hypertension, Parkinson’s Disease', Reumathoid arthritis, TET

## Introduction

The quest to understand the molecular drivers of human diseases has been steadily moving beyond the genetic sequence itself, increasingly turning towards the intricate molecular mechanisms that govern its regulation. In this light, DNA methylation acts as the critical software that executes precise cellular programs within the genome’s hardware, and, when corrupted, orchestrates the onset of diverse disease states.

Reflecting the *momentum* in this field, this Research Topic in Genome Organization and Dynamics brings together a total of six articles about “Discussing the implication of DNA methylation in human diseases”. Two comprehensive Review papers explore the role of the DNA demethylation initiator, Ten-Eleven Translocation 2 (TET2), in both disease aetiology and potential therapeutic strategies, as well as the growing clinical importance of epigenetic clocks in tracking and influencing age-linked conditions. Rounding out the collection, four original Research articles uncover novel methylation signatures across different human pathologies, from Parkinson’s Disease (PD) to hypertension, opening also to the importance of epigenetics in cell-free (cf) DNA in chronic diseases like rheumatoid arthritis (RA).

Taken together, these contributions address the broad relevance that DNA methylation has to human disease and converge on a common theme: the dynamism of this epigenetic modification. DNA methylation, indeed, is not a static feature of the genome but, rather, a dynamic layer that adapts in response to developmental environment and cellular cues, profoundly affecting cellular function and identity. It is not surprising, then, that any alteration drives from a physiological function to pathological consequences.

Keeping a correct DNA methylation status ensures not simply gene expression profiles, but also more widely genome stability. Consequently, disturbances in these patterns can contribute to aging and disease through inappropriate silencing or activation of genes, altered chromatin states, genomic instability, or disruption of developmental programs, so that DNA methylation has been considered as a health predictor (Xu, 2025). Depending on the specific context, though, changes in DNA methylation may be initiator of events or consequences of already altered cellular states.

Based on the above premises, the present collection can be divided into three subtopics, specifically addressing: 1) TET2 functions and its implications in human diseases, 2) DNA methylation and the epigenetic clock and 3) DNA methylation patterns in human inflammatory states and pathologies.

## TET2 function and its implications in human disease

Despite the long-standing understanding of DNA methylation, its counterpart, active DNA demethylation, involves complex multi-step enzymatic cascades whose precise fine-tuning is still under investigation. The activity of TET family of enzymes acts as the primary mediator of this process (Carollo and Barra, 2025). In this regard, Tang et al. here focus their attention specifically on a member of the TET family, TET2, which is known to catalyse the first step of active DNA demethylation by oxidizing 5-methylcitosine (5 mC) to 5-hydroxymethylcytosine (5hmC). Wild type TET2 maintains cellular physiology and genetic stability, whereas TET2 somatic mutations are implied in several pathologies, ranging from inflammatory disorders to cancer. Therefore, understanding TET2 function in the pathological contexts can be harnessed to develop promising therapeutic strategies (Tang et al.).

## DNA methylation and the epigenetic clock

DNA methylation changes have also been associated with ageing. The term “epigenetic clock”, introduced for the first time by Steve Horwath in 2013, indeed correlates DNA methylation pattern variations within human tissues and cell types with their “ageing” phenotypes (Horvath, 2013). Since then, different “epigenetic clocks” have risen, and it is now possible to apply them not only to tissues, but also to populations and pathological contexts. In their review, Chen et al. dissect the basis of DNA methylation as well as progresses in the epigenetic clock and their following implications in ageing research, health control and disease prediction (Chen et al.).

## DNA methylation patterns in human inflammatory states and pathologies

Specific DNA methylation patterns can be predictive of pathological conditions, as found for RA. Indeed, changes in DNA methylation in the *DUSP22* gene have been associated with RA and erosive disease. In this regard, Delgado-Cruzata et al., by using pyrosequencing on plasma cfDNA samples from 27 RA patients, found that these patients displayed lower cfDNA *DUSP22* DNA methylation at one specific DNA methylation site in the promoter region compared to controls. In addition, decreased methylation levels on cfDNA are associated with increased joint space narrowing. Thus, cfDNA *DUSP22* DNA methylation may be considered as a new, non-invasive biomarker for RA (Delgado-Cruzata et al.).

Also, hypertension can be predicted via DNA methylation pattern analysis. Indeed, in their research paper, Zhu et al., by using Illumina Infinium Methylation EPIC BeadChips, analyzed the epigenome-wide methylation profile of peripheral leukocytes extracted from 179 patients with hypertension and found an association between alterations of DNA methylation patterns with inflammation biomarkers (Zhu et al.).

Variation of DNA methylation patterns can also be implied in the regulation of inflammatory cytokine expression, as found by Sankar et al. in newborns with late-onset neonatal sepsis (LOS). Infants with LOS, indeed, display a pattern characterized by hypomethylated pro-inflammatory genes and hypermethylated anti-inflammatory genes, compared to their healthy counterpart. Thus, expression of inflammatory genes in LOS, such as *TNF-α*, *IFN-γ*, *IL-10*, and *TGF-β*, is influenced by DNA methylation. Therefore, this new knowledge can result in both improved diagnostics and therapy to treat LOS (Sankar et al.).

DNA methylation profiles cannot only be used as disease predictive biomarkers, but also to analyze the incidence of a pathology according to sex and geographical location in a specific ethnic group. To ascertain methylation variations at CpG sites within the PD related genes *MAPT* and *SNCA* in the Mexican Mestizo population, Miranda-Morales et al. performed bisulfite pyrosequencing on leukocyte DNA from PD patients and age and sex matched healthy counterpart. In PD patients, the authors found reduced methylation in the promoter region of *MAPT* gene and in the intron one of *SNCA* gene as well. Interestingly, differences in DNA methylation also depended on the regional location as well as on the sex of the patients. Specifically, men displayed significant difference in *MAPT* gene methylation, while woman accounted for significant methylation differences in *SNCA* gene. Therefore, sex and geographical specific DNA methylation patterns in PD can be established (Miranda-Morales et al.).

## Concluding remarks

By combining multi-omic and clinical data, DNA methylation research is evolving from individual locus identification to an integrated view of disease biology. This integration helps clarifying the mechanisms underlying disease heterogeneity and uncovers molecular signatures that can have values from multiple points of view: diagnostics, prognosis, and therapy as well. Indeed, the reversible nature of DNA methylation offers a promising avenue for therapeutic targeting of aberrant methylation patterns.
